# Post-Seismic Deformation from the 2009 Mw 6.3 Dachaidan Earthquake in the Northern Qaidam Basin Detected by Small Baseline Subset InSAR Technique

**DOI:** 10.3390/s16020206

**Published:** 2016-02-05

**Authors:** Yang Liu, Caijun Xu, Yangmao Wen, Zhicai Li

**Affiliations:** 1 School of Geodesy and Geomatics, Wuhan University, Wuhan 430079, China; cjxu@sgg.whu.edu.cn (C.X.); ymwen@sgg.whu.edu.cn (Y.W.); 2 Key Laboratory of Geospace Environment and Geodesy, Ministry of Education, Wuhan University, Wuhan 430079, China; 3 Collaborative Innovation Center for Geospatial Technology, Wuhan 430079, China; 4 Department of Geodesy, National Geomatics Center of China, Beijing 100048, China; zcli@nsdi.gov.cn

**Keywords:** post-seismic deformation, the 2009 Mw 6.3 Dachaidan earthquake, the Qaidam basin, small baseline subset technique, InSAR

## Abstract

On 28 August 2009, one thrust-faulting Mw 6.3 earthquake struck the northern Qaidam basin, China. Due to the lack of ground observations in this remote region, this study presents high-precision and high spatio-temporal resolution post-seismic deformation series with a small baseline subset InSAR technique. At the temporal scale, this changes from fast to slow with time, with a maximum uplift up to 7.4 cm along the line of sight 334 days after the event. At the spatial scale, this is more obvious at the hanging wall than that at the footwall, and decreases from the middle to both sides at the hanging wall. We then propose a method to calculate the correlation coefficient between co-seismic and post-seismic deformation by normalizing them. The correlation coefficient is found to be 0.73, indicating a similar subsurface process occurring during both phases. The results indicate that afterslip may dominate the post-seismic deformation during 19–334 days after the event, which mainly occurs with the fault geometry and depth similar to those of the c-seismic rupturing, and partly extends to the shallower and deeper depths.

## 1. Introduction

**Figure 1:**
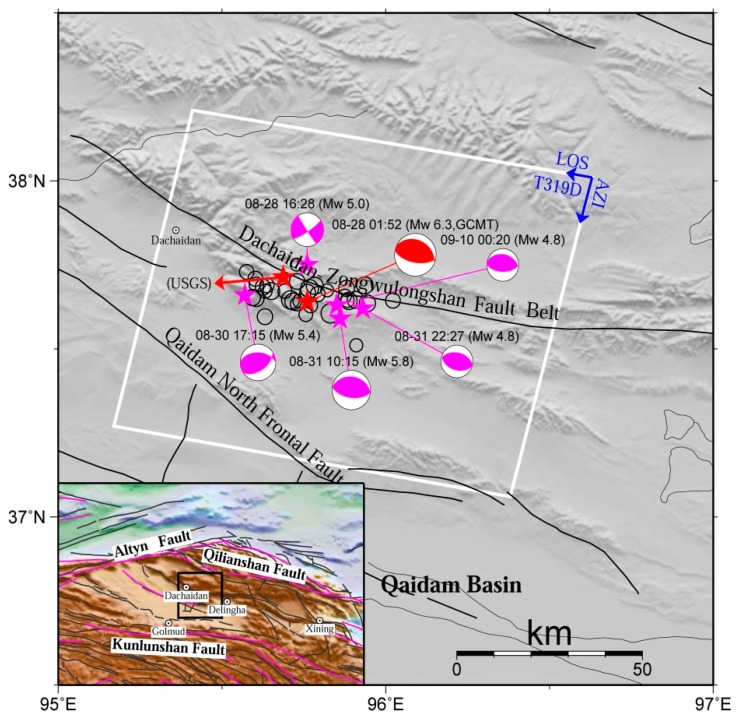
Tectonic map showing the 2009 Mw 6.3 Dachaidan earthquake region. The extent of the adopted Envisat ASAR data (descending Track 319) is delimited by the white rectangle, with LOS and AZI denoting radar line-of-sight (LOS) and azimuth directions. The Global Centroid Moment Tensor (GCMT) focal mechanisms of the main shock and the five aftershocks are displayed by red and purple beach balls, respectively, and the other aftershocks are displayed by black circles. The United States Geological Survey (USGS) epicenter of the main shock is also displayed. The active tectonic faults from Deng *et al.* [9] and Peltzer and Saucier [11] are displayed by black and purple lines, respectively.

The 28 August 2009 Mw 6.3 Dachaidan earthquake [1,2,3,4,5] in the northern Qaidam basin, China, was the second Mw 6.3 event along the northern Qaidam basin fault system between 2008 and 2009. The first Mw 6.3 event occurred a few kilometers away from the 2009 event’s epicenter on 10 November 2008 [6,7]. In addition, another Mw 6.3 event on 17 April 2003 happened tens of kilometers to the east of the 2009 event’s epicenter [4,8]. The earthquake zone is located in the northern region of the Qaidam basin block (Figure 1), which is bounded by the Altyn fault to the northwest, the Qilianshan fault to the northeast, and the Kunlunshan fault to the south [9,10]. These seismogenic behaviors and tectonic characteristics provide an opportunity to investigate the evolution of the earthquake process in the region. To achieve this, it is essential to understand, in-depth, the rupturing and deformation mechanism of the existing events during the co-seismic and the post-seismic phases using multi-disciplinary methods, such as traditional seismological and modern geodetic techniques.

It is well known that high-precision and high-spatial resolution co-seismic displacement from geodetic techniques, such as Interferometric Synthetic Aperture Radar (InSAR) and Global Positioning System (GPS) methods, is critical for understanding the static co-seismic rupturing mechanism of the underground fault regime [12,13,14,15,16,17,18,19,20,21,22,23,24]. In addition, post-seismic studies of tens of strong and large (≥Mw 6) earthquakes around the world indicated that post-seismic deformation can play an important role in a detailed determination of the post-seismic response to an earthquake and the accurate assessment of the future seismic risk [25,26,27,28,29,30,31,32,33,34,35,36].

For the 2009 Mw 6.3 Dachaidan event, previous studies have mainly focused on the extraction of the co-seismic deformation and the determination of the static rupturing model [1,5]. Elliott *et al.* [1] investigated the co-seismic rupturing model with descending and ascending Envisat Advanced Synthetic Aperture Radar (ASAR) images, while Liu *et al.* [5] refined the rupturing model by carefully inverting the co-seismic deformation for the fault-related parameters. On the post-seismic deformation related to this event, Feng [37] derived it using seven radar images, and directly inverted the post-seismic slip by assuming the same fault geometric parameters as co-seismic rupture. However, Feng [37] did not prove the validity of this hypothesis. The aim of this study is to extract the post-seismic deformation series, followed by analyzing their spatial correlations with the co-seismic deformation and the fault rupture geometry. Thus, this study can compensate for the existing shortcoming.

GPS measurements can detect the three dimensional surface displacement related to the earthquake faulting, but its spatial coverage is always limited and sparse around the epicentral region [14,19,38]. According to Chen *et al.* [4], very few GPS stations existed around the 2009 event’s epicenter. The nearest continuous station, Xiao Qaidam station, is located at about 40 km to the southwest of the epicenter. InSAR measurements can compensate for the aforementioned limitations of sparse spatial observations of GPS technique due to their high spatial resolution. In addition, they have the advantages of unattended operation and low cost [15,38,39,40,41,42]. In this study, a total of eight images from the C-band Envisat ASAR sensor can be used. These are exploited in order to investigate the post-seismic deformation of the 2009 Mw 6.3 Dachaidan event.

In this paper, the eight Envisat ASAR images following the 2009 Mw 6.3 Dachaidan event are processed using the Small BAseline Subset (SBAS) InSAR method, with the goal of extracting the post-seismic deformation time series. We then calculate and analyze the correlation between post-seismic and co-seismic deformation using a normalization method proposed in this study. Finally, together with the co-seismic rupturing model in Liu *et al.* [5], we qualitatively interpret those post-seismic deformation observations based on the post-seismic movement mechanisms discussed over almost three decades.

## 2. Small Baseline Subset InSAR Time Series Method

Interferometric deformation field from standard InSAR processing in ROI_PAC [43] or GAMMA [44] softwares may usually be affected by the atmospheric path delay, orbital error, Digital Elevation Model (DEM) error, and other thermal noise errors [45]. The SBAS InSAR time series method, developed by Berardino *et al.* [46], can overcome these limitations of standard InSAR processing. This method has been adopted to extract the surface deformation following strong and large earthquakes around the world [34,47].

For a given interferogram, considering the effect of the DEM error, atmospheric path delay, orbital error, and other thermal noise errors, the observed InSAR phase can be expressed as [46,47,48]:

(1) $$\phi =\phi_{defo}+\phi_{\Delta_{topo}}+\phi_{APS}+\phi_{orbital}+\phi_{noise}$$

where $\phi_{defo}$ is the deformation phase along the LOS direction between master and slave image acquisition time, $\phi_{\Delta_{topo}}$ is the phase resulted from the DEM error, $\phi_{APS}$ is the phase resulted from the atmospheric delay differences between master and slave images, $\phi_{orbital}$ is the phase resulted from the orbital error, and $\phi_{noise}$ is the phase resulted from other thermal noise errors. The five terms on the right side of Equation (1) can be expressed as:

(2) $$\phi_{defo}=-\frac{4\pi }{\lambda }\left(d_{m}-d_{s}\right)$$

(3) $$\phi_{\Delta_{topo}}=-\frac{4\pi }{\lambda }\frac{B_{⊥}}{\rho \sin\theta }\Delta h$$

(4) $$\phi_{APS}=-\frac{4\pi }{\lambda }\left(d_{APS_{m}}-d_{APS_{s}}\right)$$

(5) $$\phi_{orbital}=-\frac{4\pi }{\lambda }\left(d_{orbital_{m}}-d_{orbital_{s}}\right)$$

(6) $$\phi_{noise}=-\frac{4\pi }{\lambda }\left(d_{noise_{m}}-d_{noise_{s}}\right)$$

where $d_{m}$ and $d_{s}$ are the ground positions of one pixel along the LOS direction at master and slave image acquisition time, λ, $B_{⊥}$, ρ, θ, and Δh are the radar wavelength, perpendicular baseline between master and slave image acquisitions, slant range from the satellite synthetic aperture radar (SAR) sensor to the ground point target, incidence angle, and DEM error, $d_{APS_{m}}$ and $d_{APS_{s}}$ are the atmospheric-related displacements along the LOS direction at master and slave image acquisition time, $d_{orbital_{m}}$ and $d_{orbital_{s}}$ are the orbital-error-related displacements along the LOS direction at master and slave image acquisition time, and $d_{noise_{m}}$ and $d_{noise_{s}}$ are the thermal-noise-related displacements along the LOS direction at master and slave image acquisition time.

In order to avoid large discontinuities in deformation time series, the most frequently used method is to represent the deformation term ($\phi_{defo}$) in terms of the deformation rate ($v_{k,k+1}$) between adjacent acquisition times [47]. The deformation term can be then expressed as:

(7) $$\phi_{defo}=-\frac{4\pi }{\lambda }\left(d_{m}-d_{s}\right)=-\frac{4\pi }{\lambda }\sum_{k=t_{s}}^{t_{m}-1}v_{k,k+1}\left(t_{k+1}-t_{k}\right)$$

In this case, deformation time series can be calculated by using a least squares method in the phase inversion [46,47,48]. During the inversion, pixels with insufficient observations are assigned with no value. In order to obtain the deformation field between the main shock and the first SAR acquisition time, a linear displacement velocity is assumed to extrapolate the observations backward [31,35].

## 3. Data Source and Processing

### 3.1. Data Source

Originally, both descending and ascending track SAR images from the Envisat satellite were collected, in order to obtain the post-seismic deformation series of the 2009 Mw 6.3 Dachaidan event comprehensively. Unfortunately, according to the SAR data record from European Space Agency (ESA), only two images for the ascending Track 455 were observed on 30 October 2009 and 8 January 2010, respectively. As a result, the time series surface deformation after the main event from two different viewing geometries was not obtained. Ultimately, a total of eight SAR images from descending Track 319 can be used for the InSAR time series processing (Table 1).

**Table 1:** SAR data from Envisat descending Track 319.

	Date (yyyymmdd)	Track No.	T ^a^ (days)	B_perp_^b^ (m)	Σ ^c^ (cm)	*L* ^d^ (km)
1	20090916	39451	19	0	0.07	4.72
2	20091021	39952	54	−556.9	0.20	4.72
3	20091230	40954	124	−672.9	0.36	4.72
4	20100310	41956	194	−526.2	0.36	4.24
5	20100414	42457	229	−184.8	0.35	4.40
6	20100519	42958	264	−247.5	0.34	4.84
7	20100623	43459	299	−386.9	0.37	5.56
8	20100728	43960	334	−708.1	0.46	6.12

^a^ Number of days with respect to the 28 August 2009; ^b^ Perpendicular baseline relative to the first SAR image of 16 September 2009; ^c^ Standard deviation of the interferometric deformation’s noise; ^d^ Spatial scale of the interferometric deformation’s noise.

For the available descending Track 319, the time intervals of these eight images range from 16 September 2009 to 28 July 2010. The first and last images were acquired at 19 and 334 days after the main event, respectively. The average temporal sampling for these eight images is 45 days. According to Figure 1, the available descending Track 319 can cover the whole rupturing area of the 2009 Mw 6.3 Dachaidan event. Note that compared with seven images in Feng [37], an additional image acquired on 28 July 2010 was used.

### 3.2. Data Processing

The data processing approach mainly involves two stages: interferometric processing and SBAS time series processing. The first stage aims to generate a series of interferograms, which should meet certain requirements of temporal and spatial baselines. The second stage aims to obtain the relative true deformation phase, by estimating and removing the phases related to the DEM errors, atmospheric path delays, orbital errors, and other thermal noise errors.

**Figure 2:**
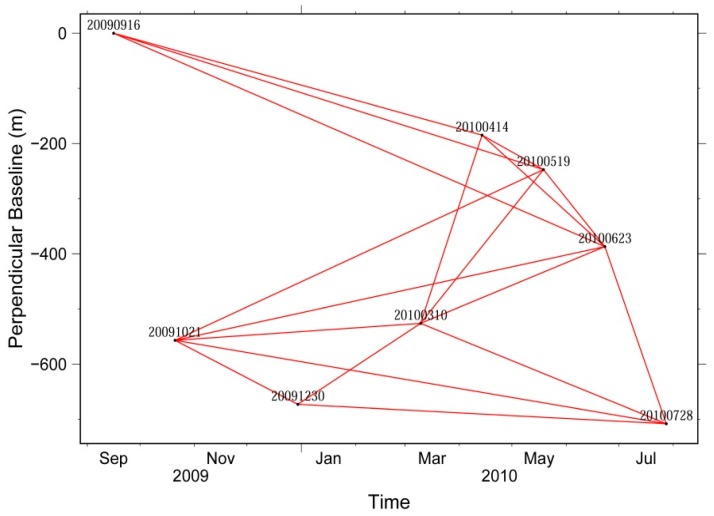
Temporal and spatial baseline for the Envisat ASAR Track 319 images.

In the interferometric processing, the available interferometric pairs were first selected, which should have a perpendicular baseline component at the scene center of less than 400 m in order to mitigate the possible spatial decorrelation and minimize the topography-induced errors [39,45]. In this case, a total of 18 interferograms can be generated, and the temporal and spatial baselines are shown in Figure 2. The interferometric pair 20090916–20100623 has the maximum perpendicular baseline of about 387 m. As shown from the Figure 2, all the baselines are interconnected together, thereby improving the stability and reliability of the parameter solutions in the following SBAS time series processing.

After the selection of the interferometric pairs, the main interferometric processing steps were conducted using the Caltech/JPL ROI_PAC software [43] by: (1) correcting the topographic phases with the three arc-second Shuttle Radar Topography Mission (SRTM) DEM [49]; (2) correcting the orbital phases with the precise orbits of ESA; (3) filtering the differential interferograms with the power spectrum filtering technique [50]; (4) unwrapping the filtered interferograms with the branch cut method [51]; and (5) transforming the unwrapped interferograms into the geodetic coordinate system. It should be noted that all the interferograms were co-registered and geocoded to a single image.

**Figure 3:**
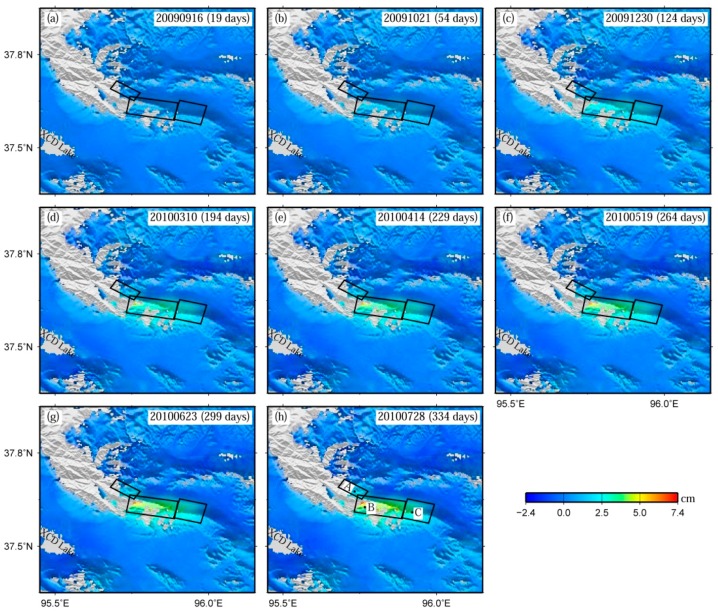
Post-seismic deformation time series of the 2009 Mw 6.3 Dachaidan event (relative to the occurrence date of the 2009 Mw 6.3 Dachaidan event, namely, 28 August 2009). Three rectangles in all subfigures display the three fault segments (the western, central, and eastern parts) used to derive the slip distribution model of the 2009 Mw 6.3 Dachaidan event [5]. A, B, and C shown in subfigure (h) are three characteristic points in Figure 4 selected to display the time series displacements close to the earthquake rupturing area. Negative values denote motion away from the satellite in the LOS direction, and positive values denote motion toward the satellite. (**a**) 20090916; (**b**) 20091021; (**c**) 20091230; (**d**) 20100310; (**e**) 20100414; (**f**) 20100519; (**g**) 20100623; (**h**) 20100728.

In the SBAS time series processing, all the interferograms were corrected for the residual orbital phases by applying the one-degree orbital polynomial fitting to the far-field phase observations, and for the atmospheric phases by filtering in both spatial and temporal domains. The deformation series were solved in the least squares sense [47,48,52]. The finally-derived post-seismic deformation time series are shown in Figure 3. Note that the displacements during the initial 19 days after the earthquake are linearly extrapolated from the displacements during days 19–54.

It should be noted that before the SBAS time series processing a highly important step is to identify the major phase unwrapping errors remaining in the interferograms using the phase closure technique [34,52,53]. Without this procedure, the derived deformation series would have a larger error. In this study, 16 phase closure interferogram pairs were generated and used to identify the unwrapping errors.

## 4. Results and Discussions

### 4.1. Temporal Features of Post-Seismic Deformation

Figure 3 shows the post-seismic displacement time series of the 2009 Mw 6.3 Dachaidan event along the LOS direction between 16 September 2009 and 28 July 2010. Compared with those from Feng [37], Figure 3 has a similar deformation trend, but retains more observations around the region near the epicenter. From the time series map, the zone to the south of the fault (the hanging wall) has an obvious pattern with a positive LOS range change, the magnitude of which gradually increases with the post-seismic time. The zone to the north of the fault (the footwall), however, has a very weak deformation pattern with a negative LOS range change. Note that negative values denote motion away from the satellite in the LOS direction, and positive values denote motion toward the satellite.

**Figure 4:**
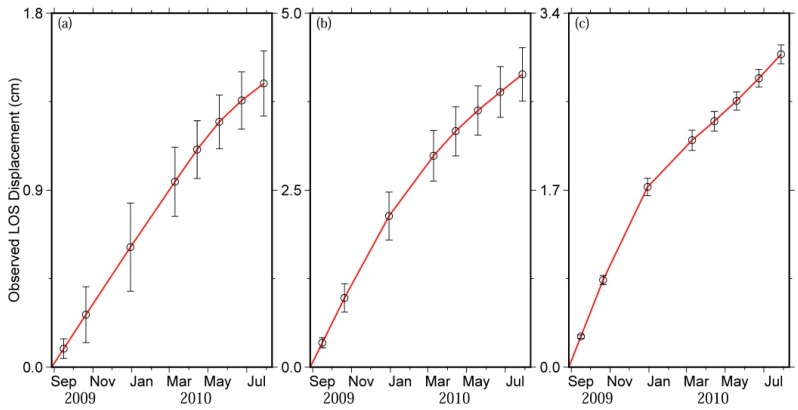
Time series displacements of three characteristic points A–C. The locations of these three points are displayed in Figure 3h. (**a**) Point A; (**b**) Point B; (**c**) Point C.

Three characteristic points, named as points A–C, are chosen to demonstrate the time series displacements close to the earthquake rupturing area (Figure 4). These points are located at the surface projection of the three fault segments of the co-seismic rupturing model from Liu *et al.* [5], respectively, and the corresponding locations are displayed in Figure 3h.

According to Figure 4, the zones around points A–C experience significant surface uplift along the LOS direction between days 19 and 334 after the Mw 6.3 earthquake. During 19–334 days after the event, the LOS range changes of the zones around points A–C increase from 0.09 cm to 1.44 cm, from 0.34 cm to 4.14 cm, and from 0.29 cm to 3.0 cm, respectively.

In addition, the changing trends of the surface deformation around these three points during 19–334 days after the Mw 6.3 earthquake are analyzed. With the initial rapid increase, the LOS range changes around all three points exhibit a decelerating trend (Figure 4). Likewise, Ryder *et al.* [32] and Bie *et al.* [35] also found a similar pattern when investigating the 2008 Nima-Gaize earthquake and the 2008 Mw 6.3 Damxung earthquake using InSAR observations.

### 4.2. Spatial Features of Post-Seismic Deformation

**Figure 5:**
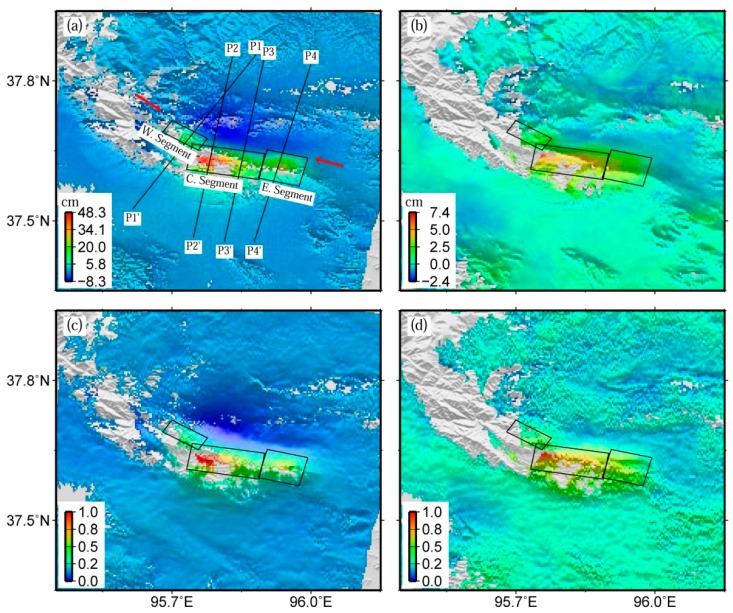
Co-seismic displacement field of the 2009 Mw 6.3 Dachaidan event (**a**); post-seismic displacement field at 334 days after the 2009 event (**b**); and the normalized co-seismic (**c**) and post-seismic (**d**) ones. P1-P1´, P2-P2´, P3-P3´, and P4-P4´shown in subfigure (**a**) indicate four profiles in Figure 6 selected to display the correlation among co-seismic deformation, post-seismic deformation, and co-seismic rupturing fault. In subfigures (**a**) and (**b**), negative values denote motion away from the satellite in the LOS direction, and positive values denote motion toward the satellite.

Figure 3h shows the post-seismic displacement map at 334 days after the 2009 Mw 6.3 Dachaidan event. It can be seen from Figure 3h that the LOS range change displays a pattern of positive values to the south of the fault (the hanging wall) with a maximum uplift up to 7.4 cm, and weak negative values to the north (the footwall). The maximum uplift up to 7.4 cm is close to that (~7 cm) of Feng [37]. The deformation magnitude to the south is significantly greater than that to the north. This difference across the co-seismic rupturing fault line is consistent with the pattern of the co-seismic deformation field in Figure 5a (See Section 4.3 and Section 4.4 for detailed comparisons).

The LOS range changes around the main deformation zone of the hanging wall are then analyzed (Figure 3h and Figure 4). The zone around point B has the largest deformation of up to 4.14 cm, followed by the zone around point C with a deformation of 3.0 cm, and the zone around point A has a deformation of only 1.44 cm. Considering the spatial distribution of these three points (Figure 3h), one can conclude that the significant deformation is located at the central part of the hanging wall, and the deformation has an overall decreasing trend from the central part to the western and eastern ones. This spatial characteristic of the post-seismic deformation across the hanging wall is also consistent with that shown in the corresponding co-seismic deformation field (Figure 5a).

The noise properties of each derived deformation interferogram are investigated by using a one-dimension covariance function. This method has been extensively adopted to calculate the corresponding noise magnitude and spatial scale [5,22,54]. During the calculation, only observations far from the obvious post-seismic deformation area are used to fit the one-dimension covariance functional model. The calculated magnitude and spatial scale of errors are displayed in Table 1. The magnitudes of errors are in the range from 0.07 cm to 0.46 cm, while from 4.24 km to 6.12 km, for the spatial scale.

### 4.3. Correlation between Post-Seismic and Co-Seismic Deformation

The earthquake deformation cycle can be typically divided into three phases, named as co-seismic deformation, post-seismic deformation, and inter-seismic deformation [55,56]. The co-seismic deformation instantaneously occurring during an earthquake is followed by a post-seismic deformation with transient pattern, which finally decays to an inter-seismic deformation with a steady-state pattern. The co-seismic dislocation happens in order to compensate for the deformation accumulated during the inter-seismic phase [57], whereas the transient post-seismic process occurs as a response to the co-seismic rupturing [56]. To investigate the post-seismic deformation response to the co-seismic rupturing of the 2009 Mw 6.3 Dachaidan event, the spatial correlation between co-seismic and post-seismic deformation field is investigated. The co-seismic deformation field (Figure 5a) was derived with the Envisat descending Track 319 images acquired on 14 January 2009 and 16 September 2009, with the perpendicular baseline of about 222 m [5].

Figure 5a,c shows the spatial distribution of the original and normalized co-seismic deformation of the 2009 event, and Figure 5b,d for the original and normalized post-seismic deformation at 334 days after the 2009 event. The normalized deformation is calculated by rescaling maximum and minimum values to 1 and 0, respectively. It is clear in Figure 5 that both original and normalized post-seismic deformation fields display a similar changing pattern with the corresponding co-seismic deformation, qualitatively indicating a high spatial correlation between them.

Based on the normalized co-seismic and poseismic deformation observations, further, the spatial correlation coefficient between them can be calculated. The coefficient is found to be 0.73, quantitatively indicating a strong spatial correlation between co-seismic and post-seismic deformation. The comparability of co-seismic and post-seismic deformation field potentially indicates that a similar underground process happened during the co-seismic and the observed post-seismic phases. Likewise, in the co-seismic and post-seismic deformation study of the 2008 Nima-Gaize earthquake in the Tibet Plateau, Ryder *et al.* [32] extracted the co-seismic and post-seismic interferograms and found their first-order similarities, and then inferred that the same subsurface process occurred during both co-seismic and observed post-seismic phases.

### 4.4. Correlation between Post-Seismic Deformation and Co-Seismic Rupturing Fault

Section 4.3 suggests that a strong correlation exists between co-seismic and post-seismic deformation fields. It is well known that co-seismic deformation observations can be predicted from earthquake faulting using an elastic dislocation model [58,59]. By examining a large number of earthquake cases, post-seismic deformation may be controlled by the co-seismic rupturing fault, in the form of afterslip, or poroelastic rebound, or viscoelastic relaxation, or their combinations during the post-seismic phases [27,34,35,56]. In this section we will investigate the correlation between the post-seismic deformation and co-seismic rupturing fault, and qualitatively interpret the post-seismic deformation mechanism.

**Figure 6:**
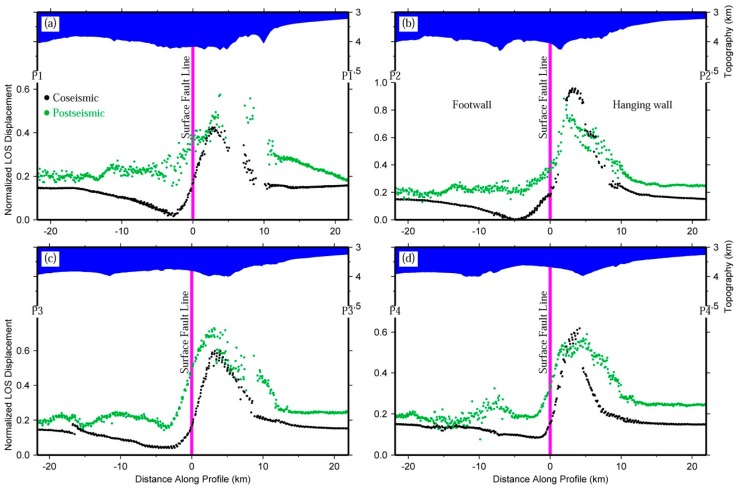
Normalized co-seismic deformation, post-seismic deformation, and topography along profiles (**a**) P1-P1´; (**b**) P2-P2´; (**c**) P3-P3´; and (**d**) P4-P4´. The locations of these four profiles are displayed in Figure 5a. The purple vertical bars denote the locations of the surface fault lines crossed over by the corresponding profile.

Figure 5a,c shows the three rupturing fault segments used to derive the slip distribution model of the 2009 Mw 6.3 Dachaidan event [5]. It can be seen that the pattern of the LOS range displacements changes with the geometric shape of the fault model in both co-seismic and post-seismic deformation. Figure 6 shows four normalized post-seismic and co-seismic displacement profiles across the surface fault line, with profile P1-P1´ crossing the western segment of the co-seismic rupturing fault, P2-P2´ and P3-P3´ crossing the central one, and P4-P4´ crossing the eastern one. According to Figure 6, the spatial wavelength of post-seismic deformation is generally larger than that of co-seismic deformation, whereas it is slightly shorter in profile P2-P2´ (Figure 6b). It should be noted that the small undulations are not relevant because it is difficult to discriminate on the post-seismic deformation. In addition, the normalized values of post-seismic deformation are relatively larger than those of co-seismic deformation for the zone close to the surface fault line.

These aforementioned facts may imply that the post-seismic transient process during the observed period is dominated by the similar fault geometry as the co-seismic slip, that this process primarily occurs at a comparable depth to the co-seismic process, and also partly extends to the shallower and deeper depths. The time-dependent change characteristics of this post-seismic transient process can be obtained from the investigation of the post-seismic deformation time series (Figure 3 and Figure 4). Comparison of the deformation series ranging from 19–334 days after the event suggests that the post-seismic transient process may have a decelerating trend.

In light of the temporal and spatial characteristics of three post-seismic mechanisms, one can speculate that the observed post-seismic deformation during 19–334 days after the Mw 6.3 earthquake should be closely related to the afterslip mechanism. This distribution potentially reveals a co-seismic slip deficit, which is compensated by post-seismic afterslip and/or inter-seismic creep associated with the co-seismic rupturing fault. This phenomenon has been found in the study of other earthquake cases, such as the 2003 Mw 6.8 Chengkung, Taiwan, earthquake [60].

## 5. Conclusions

This study uses the SBAS InSAR technique to extract the post-seismic deformation time series of the 28 August 2009 Mw 6.3 Dachaidan event, situated at the northern Qaidam basin, China. The derived post-seismic deformation has a decelerating trend during the 19–334 days after the 2009 Mw 6.3 earthquake, with a maximum uplift up to 7.4 cm along the LOS direction at 334 days after the event. The significant deformation region across the observing area is located at the hanging wall, where the deformation has a decreasing trend from the central part to the western and eastern ones.

Based on the normalization processing, a calculation method of correlation is proposed and used to determine the spatial correlation between co-seismic and post-seismic deformation of the 2009 Mw 6.3 Dachaidan event in this study. We find that these two deformation fields have a correlation coefficient of 0.73. This strong correlation potentially suggests a similar underground process occurring during both co-seismic and observed post-seismic phases. The relevance analysis among post-seismic deformation, co-seismic deformation, and co-seismic rupturing fault indicates that the afterslip mechanism may dominate the post-seismic deformation during the observed time interval, and mainly occur with similar fault geometry and depth as the co-seismic rupturing. In addition, it is inferred that afterslip may partly extend to the shallower and deeper depths.

With more available observations, such as geological field investigation and optical remote sensing data, one can further accurately examine the dominant post-seismic deformation mechanisms, or even the time-varying deformation mechanisms.
